# From Vision Changes to Hospice: The Rapid Progression of Orbital Metastasis in Hepatocellular Carcinoma

**DOI:** 10.7759/cureus.80288

**Published:** 2025-03-09

**Authors:** Aamir Shaikh, Hassan Syed, Shahnawaz N Notta, T.J. Mitchell, Lance Klosterman

**Affiliations:** 1 Internal Medicine, East Tennessee State University James H. Quillen College of Medicine, Johnson City, USA; 2 Radiology, Mountain Home Veterans Affairs Medical Center, Johnson City, USA

**Keywords:** delayed cancer treatment, extrahepatic metastases, hepatocellular carcinoma, oncologic ophthalmology, orbital metastasis

## Abstract

Hepatocellular carcinoma (HCC) is an aggressive primary liver malignancy with a high potential for metastasis, commonly involving the lungs, bones, and lymph nodes. However, orbital metastasis is an exceptionally rare presentation. This case describes a 73-year-old man with a history of HCC, hypertension, type 2 diabetes with neuropathy, chronic obstructive pulmonary disease (COPD), hepatitis C, liver cirrhosis, and portal hypertension who presented with a two-week history of blurry vision in the left eye, numbness on the left forehead, and a sensation of orbital fullness. He also reported an episode of unintentional bilateral upper extremity jerking followed by nausea and vomiting. Examination revealed left-sided proptosis, and imaging identified a retrobulbar orbital mass with muscle involvement. Biopsy confirmed metastatic HCC. Despite an initial referral for Y-90 radiotherapy, the patient was unable to undergo treatment due to multiple hospitalizations for unrelated conditions. Following the diagnosis of orbital metastasis, he experienced rapid functional decline, including multiple falls, cognitive deterioration, and loss of independence in activities of daily living, ultimately requiring hospice care. This case underscores the importance of recognizing rare metastatic patterns in HCC, especially in patients presenting with ocular symptoms. It highlights the severe consequences of delayed cancer treatment and the critical role of coordinated care in managing complex patients. Clinicians must remain vigilant in identifying unusual symptoms in patients with HCC to facilitate early diagnosis and timely intervention for advanced metastatic disease.

## Introduction

Hepatocellular carcinoma (HCC) accounts for approximately 900,000 new cases a year, ranking as the sixth most commonly diagnosed cancer and third leading cause of cancer death globally [1]. HCC is an aggressive malignancy with a high propensity for vascular invasion and hematogenous spread. Vascular invasion, which can be categorized as microvascular (microscopic) or macrovascular (grossly visible), is a key prerequisite for hematogenous dissemination and is an independent predictor of poor prognosis and decreased survival [2]. The most common pattern of vascular invasion involves the portal vein, leading to complications such as portal hypertension and ascites. Tumor invasion of the hepatic veins can extend into the inferior vena cava (IVC) and right atrium, allowing tumor cells to enter the systemic circulation. This facilitates metastatic spread to distant organs, most commonly the lungs and bones.

Three primary routes of hematogenous spread in HCC have been identified: direct invasion of intrahepatic arterioles and venules, retrograde flow through the portal venous system due to portal hypertension, and collateral circulation through lumbar veins from intra-abdominal pressure increase [2]. While the lungs and bones are frequent sites of metastasis, orbital involvement is exceedingly rare. However, the rich vascular network within the orbit makes it a potential site for tumor cell deposition and growth. This unusual metastatic pattern underscores the aggressive nature of HCC and highlights the importance of recognizing atypical presentations, especially in patients with a history of vascular invasion. Early identification of such rare metastatic sites can have significant implications for timely diagnosis, management, and prognosis.

## Case presentation

A 73-year-old man with a past medical history significant for hypertension, type 2 diabetes mellitus with neuropathy, chronic obstructive pulmonary disease (COPD), hepatitis C, liver cirrhosis, portal hypertension, and HCC (diagnosed in 2022) presented to the emergency department (ED) with a two-week history of vision changes described as blurry and diminished vision in the left eye. He also reported an isolated episode of unintentional bilateral upper extremity "jerking" followed by nausea and vomiting later that same night. Additional symptoms included numbness and tingling on the left side of the forehead and a sensation of fullness in the left eye. He denied focal weakness, confusion, slurred speech, fever, or chills.

**Figure 1:**
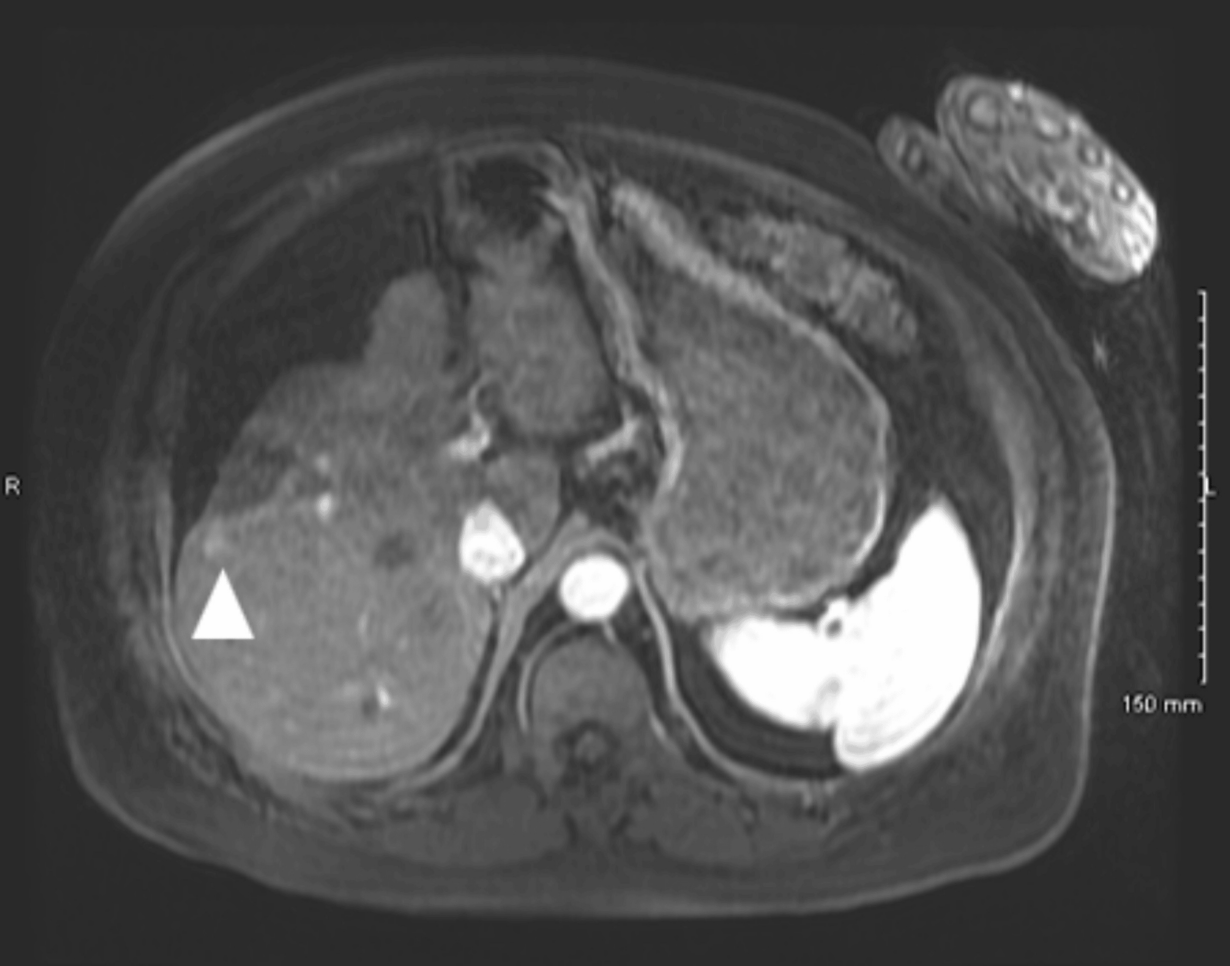
Post-microwave ablation MRI demonstrates nodular enhancement consistent with viable tumor

On examination, the patient displayed chronic facial asymmetry, which was attributed to prior plastic surgery, and left-sided proptosis. The review of systems was negative for recent trauma or infections. Since his initial HCC diagnosis (Figure 1), he had been referred to interventional radiology for Y-90 radiotherapy by Gastroenterology. However, due to repeated hospitalizations for a sacral decubitus ulcer and a pulmonary embolism, he was unable to pursue treatment and thus did not receive radiotherapy or other interventional therapies.

**Figure 2:**
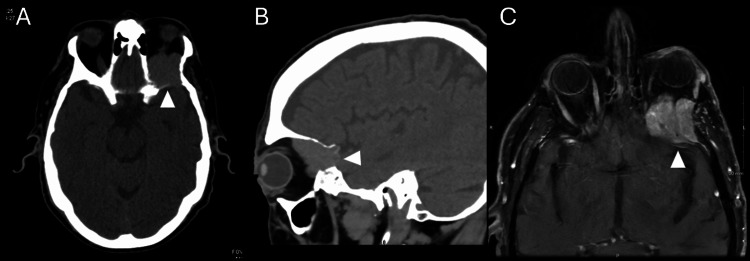
Head CT and MRI demonstrate left sphenoid bone metastasis extending into the posterior orbit

Subsequent imaging with CT and MRI in the ED revealed a left retrobulbar orbital mass with muscle involvement (Figure 2A-2C). The Ophthalmology service was consulted, and a biopsy was recommended and subsequently performed by the oculoplastics team, confirming the mass as metastatic HCC. Following the diagnosis, the patient experienced a rapid functional decline, including multiple falls, significant difficulties with activities of daily living (ADLs) and instrumental activities of daily living (IADLs), cognitive deterioration, and urinary incontinence. This progression ultimately resulted in patient placement into hospice care. 

## Discussion

HCC is the most prevalent type of primary liver cancer, accounting for approximately 75% of total cases [3]. Due to advancements in treatment and survival, there has been an increase in the identification of extrahepatic metastases, which make up about 15-17% of cases of HCC [4]. Our case observes one of the rare locations of metastasis in HCC patients, presenting in the patient's left retrobulbar orbit.

A national study done in 2014 quantified HCC metastasis in over 50,000 patients from the University Health Consortium (UHC) and Nationwide Inpatient Sample (NIS). The databases showed that 6% of patients had lung metastasis, 3% to the peritoneum, 3% to the bones, 2% to the lymph nodes, 1% to the spleen, and 1% to the adrenals, encompassing the most common locations of HCC extrahepatic metastasis [5]. In another study done in 2011 analyzing outcomes of HCC patients with extrahepatic metastases, the most common sites included the lungs (39.5%), lymph nodes (34.2%), bones (25.4%), adrenal glands (8.8%), brain (1.2%), spleen (0.6%), and breast (0.3%), for a total of 376 extrahepatic occurrences in 342 patients [6]. Recent literature suggests that HCC metastatic disease to the orbit, while not impossible, is rare compared to other extrahepatic locations.

In discovered orbital masses in the general patient population, metastases comprise an overall minimal amount of cases. A systematic review of orbital metastases done in 2021 showed that the most common sources of these malignancies are breast (36.3%), melanoma (10.1%), and prostate (8.5%) cancers [7]. Liver cancer, including HCC, only accounts for approximately 3.4% of orbital metastases, most commonly presenting with proptosis, diplopia, impaired eye motility, palpable masses, orbital pain, impaired vision, and ptosis [8]. Confirmed cases of expressly HCC orbital metastases are limited in the literature.

Evidence suggests that the presence of orbital metastasis from HCC is a poor prognostic indicator. Orbital metastasis from HCC is a relatively rare occurrence, accounting for only 1-13% of all orbital tumors and affecting approximately 2-5% of cancer patients [9]. When it does occur, prognosis is typically poor, with an average survival time of approximately 10 months, though this can vary based on the overall disease burden and status of the primary tumor [10,11]. The presence of orbital metastasis often signifies advanced, disseminated disease, indicating a higher systemic tumor burden and aggressive tumor behavior [10,11]. Notably, orbital metastasis can sometimes be the initial presenting symptom of HCC, underscoring the importance of considering HCC as a potential primary source when evaluating new orbital masses [9,12]. Although rare, orbital metastasis from HCC should be recognized as a poor prognostic factor that warrants prompt diagnosis and management, given its association with dismal overall survival [9-11]. Delays in treatment have been shown to significantly impact survival in HCC patients, with studies demonstrating that delayed treatment is associated with worse survival rates and an increased risk of mortality [13]. In summary, orbital metastasis in HCC patients is a reliable indicator of advanced, aggressive disease and is associated with a poor prognosis, highlighting the need for vigilance and timely intervention by clinicians.

## Conclusions

This case underscores several critical points for internists managing HCC patients. First, orbital metastasis, though rare, should be considered in patients with HCC presenting with new-onset ocular symptoms. Second, this case illustrates the severe consequences of delayed cancer treatment due to comorbid conditions, emphasizing the importance of coordinated, multidisciplinary care to mitigate interruptions in therapy. Finally, the rapid decline following the diagnosis of orbital metastasis highlights its association with poor prognosis, reinforcing the need for vigilance in early symptom recognition and timely management. This report highlights the need to consider metastatic disease in the differential for patients with HCC who present with ocular disturbances and the importance of comprehensive patient care.
